# Cardioprotective effect of *Rosa canina* L. methanolic extract on heat shock induced cardiomyocyte injury: An experimental study

**DOI:** 10.34172/jcvtr.2020.47

**Published:** 2020-11-28

**Authors:** Ava Nasrolahi, Leila Hosseini, Fatemeh Farokhi-Sisakht, Javad Mahmoudi, Pouran Karimi, Reza Badalzadeh, Marjan Erfani

**Affiliations:** ^1^Infectious Ophthalmologic Research Center, Health Research Institute, Ahvaz Jundishapur University of Medical Sciences, Ahvaz, Iran; ^2^Pain Research Center, Health Research Institute, Ahvaz Jundishapur University of Medical Sciences, Ahvaz, Iran; ^3^Neurosciences Research Center (NSRC), Tabriz University of Medical Sciences, Tabriz, Iran; ^4^Department of Physiology, Tabriz University of Medical Sciences, Tabriz, Iran; ^5^Higher Education Institute of Rabe-Rashid, Tabriz, Iran

**Keywords:** Rosa Canina, Oxidative Stress, Endoplasmic Reticulum Stress, Heat Stress, Cell Death

## Abstract

***Introduction:*** Overexposure to heat conditions can affect the functioning of the cardiovascular system and may promote cardiovascular disorders. Heat shock induced myocardial injury via increasing endoplasmic reticulum response-mediated apoptosis. This study investigated the impact of pretreatment with *Rosa canina* (RC), a natural antioxidant, on myocardial damage induced by heat stress exposure and underlying mechanisms in cardiomyocytes in rats.

***Methods:*** Sixty adult male Wistar rats were allocated into five groups, including Control: received normal saline (NS), Heat Stress (HS), and HS+RC groups. Animals in the HS groups were subjected to heat stress (43 ºC) for 15 minutes once a day for two weeks. Animals in the HS+RC groups received three doses of RC (250, 500, and 1000 mg/mL) one hour before being subjected to heat shock. The endoplasmic reticulum (ER) transmembrane kinases, including PKR-like endoplasmic reticulum kinase (PERK), immunoreactivity of CCAAT/enhancer-binding protein homologous protein (CHOP), and eukaryotic translation initiation factor 2-alpha (eIF2α) as well as caspase 8 were detected by Western blot. The levels of reactive oxygen species (ROS) were assessed. Moreover, histopathological changes and apoptosis were also assayed in the heart tissue by using histopathological and TUNEL assays.

***Results:*** Heat exposure increased the level of ROS and induced oxidative damage in the heart tissue. The results demonstrated that RC administration decreased the overproduction of ROS induced by heat stress in cardiomyocytes. Moreover, heat stress up regulated the expression of p-PERK, p-eIF2α,and CHOP protein while pretreatment with RC decreased expression of ER stress-related markers in cardiomyocytes. Besides, RC diminished heat stress-induced cellular damage and apoptosis associated with inhibition of caspase 8 activation, a pro-apoptotic protein in cardiomyocytes.

***Conclusion:*** These findings indicate that RC exerts a protective effect on heart tissue, at least in part,through inactivation of PERK/eIF2α/CHOP pathway or inhibition of ER stress and oxidative stress triggeredapoptosis in cardiomyocytes induced by heat stress.

## Introduction

The universal temperature is increasing over the last few years and estimated to rise even more, which means extreme exposure to heat, particularly during hot seasons in tropical countries or a desert environment.^1^ Rise in body temperature above 40°C is life-threatening which compromises the thermoregulatory capacity of the body. Prevention of heat-related mortality and morbidity depends on the body’s ability to deal with an extremely hot environment.^2,3^

Extreme heat exposure, an environmental hazard, is associated with different health problems affecting the cardiovascular, respiratory, and nervous system and causes death in severe cases.^4-6^ The occurrence of cardiac dysfunction, myocardial damage, and heart failure are among the cardiovascular complications seen in subjects exposed to heat stress.^7,8^ Cellular and molecular studies have shown that apoptosis and necrosis cell death can lead to heat stress-induced cardiomyocytedamage.^8,9^

The endoplasmic reticulum (ER) critically controls the synthesis, processing, translocation, and folding of proteins, calcium storage, and lipid synthesis.^10^ ER stress is defined as dysfunction of ER accompanied by the disturbance in the protein-folding process and the accumulation of unfolded proteins (UPs) in the ER lumen, resulting in activation of the inflammatory signaling pathway and consequent apoptotic cell death.^11,12^

Numerous biological insults, including oxidative stress, ischemia, hyperlipidemia, viral infection, and disturbance of calcium homeostasis, can initiate unfolded protein response (UPR) and ER stress.^8,10,11,13^ Evidence shows that ER stress and UPR play a crucial role in the development of cardiovascular disease.^13^ Moreover, recent studies provide evidence that disruption of Ca^+2^ homeostasis and oxidative stress triggered by heat stress contribute to ER stress-mediated apoptosis cell death seen in myocardial injury.^8,14,15^

*Rosa canina* (RC) is a traditional herb with a comprehensive list of benefits containing antioxidant, antimicrobial, anti-cancer, anti-diabetic, anti-inflammatory, and immunomodulatory effects.^16-21^ These effects are mostly attributed to its high flavonoid and phenolic contents, ascorbic acid, and carotenoids.^22^ RC has been applied for the prevention or treatment of different conditions like diabetes, gout, rheumatic diseases, kidney disorder, inflammation, memory dysfunction, and fever.^23-25^ Furthermore, the protective effects of RC against ischemia-reperfusion injuries have been previously reported in the isolated rat heart.^26^

Given the rising temperature in recent decades and its impact on cardiac health and heart function, in this study, we aimed to elucidate the effects of RC pretreatment on cardiac injury-induced by heat stress exposure and underlying mechanisms of its possible cardioprotective effects in rats.

## Materials and Methods

### Animals

Sixty adult male Wistar rats, about 3 months, weighing 250-300 g, were purchased from the Tabriz University of Medical Sciences (Tabriz, Iran). Rats were kept at transparent polypropylene cages and maintained under standard conditions with the temperature at (21.0 ± 2 ◦C, 60% humidity, and 12 h light/dark cycle. Tap water and food were provided ad libitum.

### Plant gathering and preparation of the extract

The dried fruits of RC were gathered from the northwest area of Iran and the genus and species were approved by the herbarium of the Faculty of Pharmacy, Tabriz University of Medical Sciences. The RC fruits were washed several times with water and then powdered. Then, 60 g of the powder was extracted with methanol and distilled water (1:1, v/v) in a Soxhlet apparatus for 10 h.^23^ Subsequently, the extract was filtered and then evaporated at 40 degrees C and dried. To make a solution of 250, 500, 1000 mg/mL, the acquired dried extract was liquefied in 0.9% normal saline.

### Heat stress exposure and RC administration

Following one week of adaptation, the animals were assigned to five groups randomly: control (C), heat stress + normal saline (NS), heat stress + RC250, heat stress + RC500, and heat stress + RC1000 groups. Animals in the heat stress groups received their respective treatments 1 hour before the onset of heat exposure by oral gavage and then were placed into a hot chamber (PECO Tech, Shiraz, Iran) at temperature 43 °C, 60 ± 10% humidity for 15 min/ day for two weeks.^27^ To avoid the effect of diurnal cycling on the results, all animals in heat stress groups were exposed to heat at approximately the same time each day.

Control animals were maintained at 21 ± 0.5 °C temperature and received 0.9% saline by gavage. Each day, RC was dissolved in 0.9% normal saline and given in doses of 250, 500, and 1000 mg/mL for two consecutive weeks. These doses were selected based on prior reported experiments.

### Sampling

After the last treatment, to anesthetize the animals, rats were intraperitoneally injected a ketamine/xylazine mixture (80/8 mg/kg). Then, animals were sacrificed, and the left ventricle tissues were instantly removed on ice-platform and kept at −70 degrees C for biochemical assessments.

### Biochemical analysis

#### Assessment of ROS levels

Intracellular ROS production levels in the heart tissue were measured using dichlorodihydrofluorescein-diacetate (DCFDA) dye. Briefly, isolated ventricular myocytes were incubated for 30 minutes in the dark in 40 μL DCFDA dye. After myocytes were washed, fluorescence intensity (FI) was read at the excitation wavelength= 485 nm and emission wavelength= 530 nm by a fluorescent plate reader. The resulting ROS levels were expressed as FI/mg protein.^28^

### Western blot analysis

The protein expressions of PERK, phospho (p)-PERK, eIF2α, p-eIF2α, pro-caspase 8, cleaved caspase 8, CHOP, and β -Actin were assessed in the left ventricle tissues using the Western blot method as previously described.^29,30^ In summary, the frozen heart tissues were homogenized in 100 µl ice-cold Radio Immuno Precipitation Assay (RIPA) buffer, which contained 1 mM protease inhibitor cocktail and then centrifuged at 12000 × g for 15 minutes at 4 °C. The protein concentration in the supernatant was detected using the Bradford assay kit. Next, equal protein amounts (30 µg) were separated on 12.5% SDS-polyacrylamide gel electrophoresis (SDS-PAGE) and then transferred to a polyvinylidenedifluoride (PVDF) membrane (Roche, UK). Non-specific binding reactions in the membranes were blocked in a blocking solution (bovine serum albumin (BSA) 3% in Tris-buffered saline (pH 7.5)) at room temperature for 1 h. Subsequently, the membranes were incubated overnight with diluted primary antibodies (Santa Cruz Biotechnology, U.S.A) against PERK, p-PERK, eIF2α, p-eIF2α, CHOP, pro-caspase 8, cleaved caspase 8, and β -Actin, in 1:500 concentrations. Afterward, the membrane was rinsed with PBS and incubated with goat anti-rabbit immunoglobulin G-horseradish peroxidase secondary antibody at room temperature for 2 hours. The blots were detected using enhanced chemiluminescence (ECL) detection kit (Pierce, Rockford, IL). Finally, the images of the protein bands were acquired and quantified by Image J software (version 1.62, National Institutes of Health, Bethesda, MD, USA).

### TUNEL assay

Heart tissue samples were fixed in 10% formalin solution for 1 week at room temperature. The fixed heart tissues were embedded into the paraffin and then sectioned into 5 μm sections using a microtome. Terminal deoxyuridine triphosphate biotin end-labeling (TUNEL) detection kit (Roche, Germany) was used for the detection of apoptotic cell death in the cardiomyocytes based on the manufacturer’s recommendations. Then, sections were labeled with anti-desmin antibody and counterstained with 5 μg/mL propidium iodide (PI) to visualize TUNEL-negative nuclei. Images were taken with a fluorescent microscope (OLYMPUS, Tokyo, Japan) and analyzed by Image J software. The numbers of TUNEL-positive cells from five different fields were counted.

### Histopathological examination

The heart tissue was washed and placed in 10% neutral buffered formalin solution for one week at room temperature. After embedding in paraffin, fixed cardiac tissue samples were cut into 5 µm serial sections using a microtome and mounted on slides. Subsequently, slides were stained with Hematoxylin and Eosin (H&E) according to the standard protocols and observed under a light microscope (Nikon, Japan).

### Statistical analysis

Data are analyzed using Graph Pad Prism 6.01 (Graph Pad Software Inc., La Jolla, CA, USA) software and shown as mean ± standard error of the mean (SEM). Statistical analyses between groups were performed using one-way ANOVA followed by Tukey post-hoc test. A *P* value of less than 0.05 was considered statistically significant.

## Results

### RC attenuates heat stress-induced ROS production

**Figure 1:**
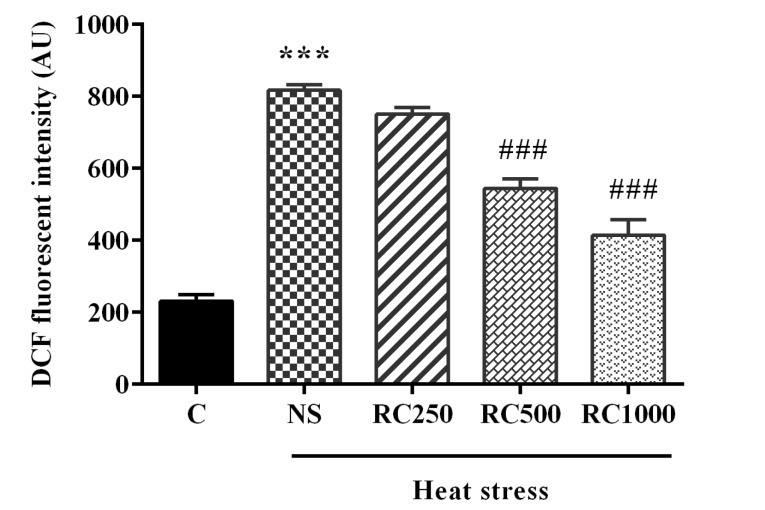
The effect of *Rosa canina* (RC) treatments on reactive oxygen species (ROS) levels in the cardiomyocytes of heat stress-exposed rats. Data are presented as mean ± SEM (n=6). ***P < 0.001 vs. C group, ###*P* < 0.001 vs. NS group. (C: control; NS: normal saline, RC: *Rosa canina*).

As Figure 1 shows, heat exposure significantly (*P* < 0.001) increased intracellular ROS levels in the heart tissue of animals compared with the control group. However, the administration of RC at doses 500 and 1000 mg/mL for two weeks markedly (*P*< 0.001 for both doses) decreased ROS levels in comparison to the NS-treated rats.

### RC inactivates PERK/eIF2α/CHOP signaling in the myocardium of heat stress-exposed rats

**Figure 2:**
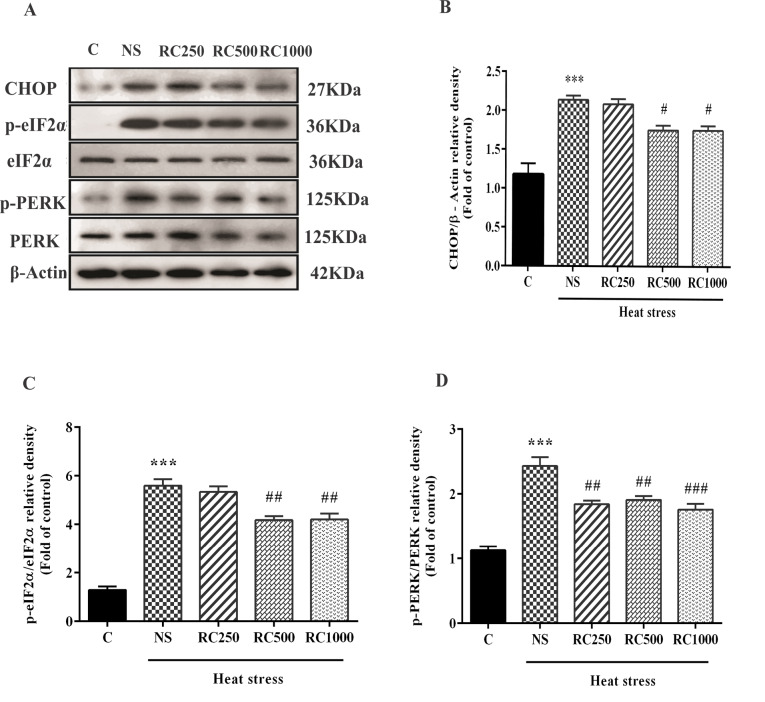
The effect of *Rosa canina* (RC) on endoplasmic reticulum (ER) stress-mediated markers in the myocardium. **A)** Immunoblotting images of PERK, p-PERK, eIF2α, p-eIF2α, CHOP, and β-actin proteins detected by Western blot. Quantitative densitometric analysis of the **(B)** p-PERK/PERK ratio, **(C)** p-eIF2α/eIF2α ratio, and **(D)** CHOP protein in study groups. Data are presented as means ± SEM (n=3): ***P < 0.001 vs. C group; #*P* < 0.05, ##*P* < 0.01, ###*P* < 0.001 vs. NS group. (C: control; NS: normal saline, RC: *Rosa canina*).

In this study, levels of p-PERK, p-eIF2α, and CHOP protein, ER stress-related markers, were determined (Figure 2). The results of immunoblotting showed that heat stress significantly increased p-PERK and p-eIF2α levels, as well as the immunoreactivity of CHOP in the ventricular tissue of NS rats when compared with the control group. Conversely, RC administration considerably decreased p-eIF2α and CHOP protein levels at doses of 500 mg/mL(*P* < 0.01 for p-eIF2α; *P* < 0.05 for CHOP) and 1000 mg/mL (*P* < 0.01 for p-eIF2α; *P* < 0.05 for CHOP), and p-PERK at doses of 250 mg/mL(*P* < 0.01), 500 mg/mL (*P* < 0.01), and 1000 mg/mL (*P* < 0.001) as compared to the NS-treated animals.

### RC decreases caspase 8 activation in the myocardium of heat stress-subjected rats

**Figure 3:**
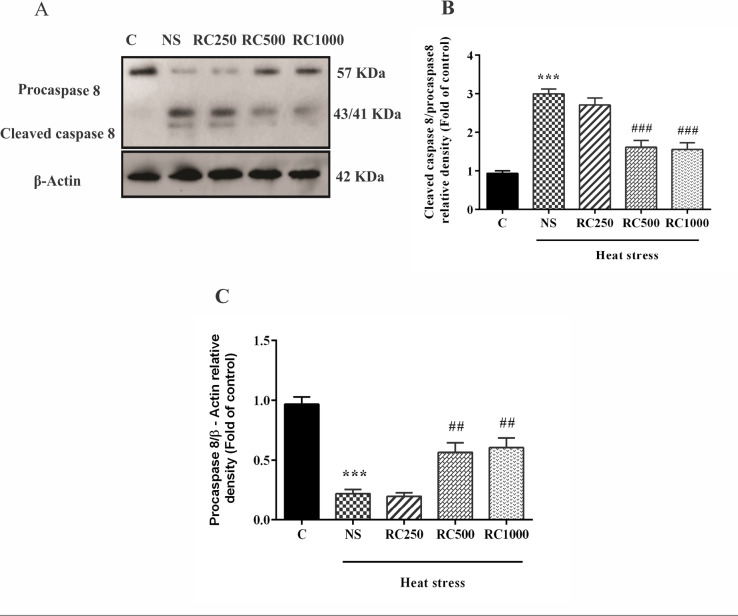
The effect of *Rosa canina* (RC) treatments on protein expression of pro-caspase 8 and its activation.** A)** Immunoblotting images of pro-caspase 8, cleaved caspase 8, and β-actin proteins detected by Western blot. Quantitative densitometric analysis of the **(B)** pro-caspase 8 and **(C)** cleaved caspase 8/pro-caspase 8 ratio in different groups. Data are presented as means ± SEM (n=3): ***P < 0.001 vs. C group; ##*P* < 0.01, ###*P* < 0.001 vs. NS group. (C: control; NS: normal saline, RC: *Rosa canina*).

As Figure 3 shows, two weeks of heat exposure significantly decreased protein expression of pro-caspase 8 (*P* < 0.001) while increased the cleavage of pro-caspase 8 (*P* < 0.001) in the ventricle tissues in comparison tothe control animals. However, protein levels of pro-caspase 8 were increased in the RC500 and RC1000 (*P* < 0.01 for both) groups. Moreover, cleaved caspase 8 was significantly decreased in the RC-received rats at doses of 500 and 1000 mg/mL (*P* < 0.01 for both doses).

### RC attenuates the number of apoptotic cell death in the cardiac myocytes of heat stress-subjected rats

**Figure 4:**
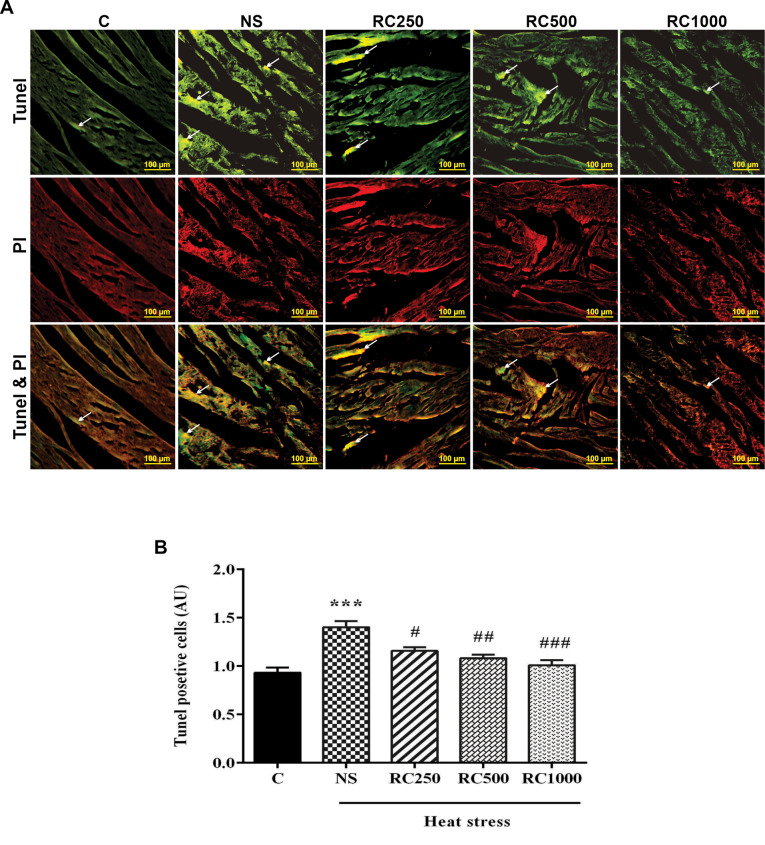
TUNEL staining of left ventricle tissues in different groups. **A)** TUNEL is shown in green and propidium iodide (PI) in red. Arrow heads show merged TUNEL-positive nuclei (green). Scale bar, 100 µm. **B)** The number of TUNEL-positive cells in the cardiac myocytes. Data are presented as means ± SEM. ***P < 0.001 vs. C group, #*P* < 0.05, ##*P* < 0.01, and ###*P* < 0.001 vs. NS group. (C: control; NS: normal saline, RC: *Rosa canina*).

Given the fact that apoptotic cell death is critically involved in heat stress-mediated cardiac dysfunction, in this study, TUNEL assay was carried to assess heart stress exposure induced cardiac damage. The results demonstrated that the number of TUNEL-positive cells in the cardiac myocytes were markedly increased in the heat stress-exposed animals (*P* < 0.001, Figure 4). By contrast, RC treatment dose-dependently attenuated heat stress-induced cell death compared to the NS-received rats.

### RC decreases the heat stress-induced cardiac injury

**Figure 5:**
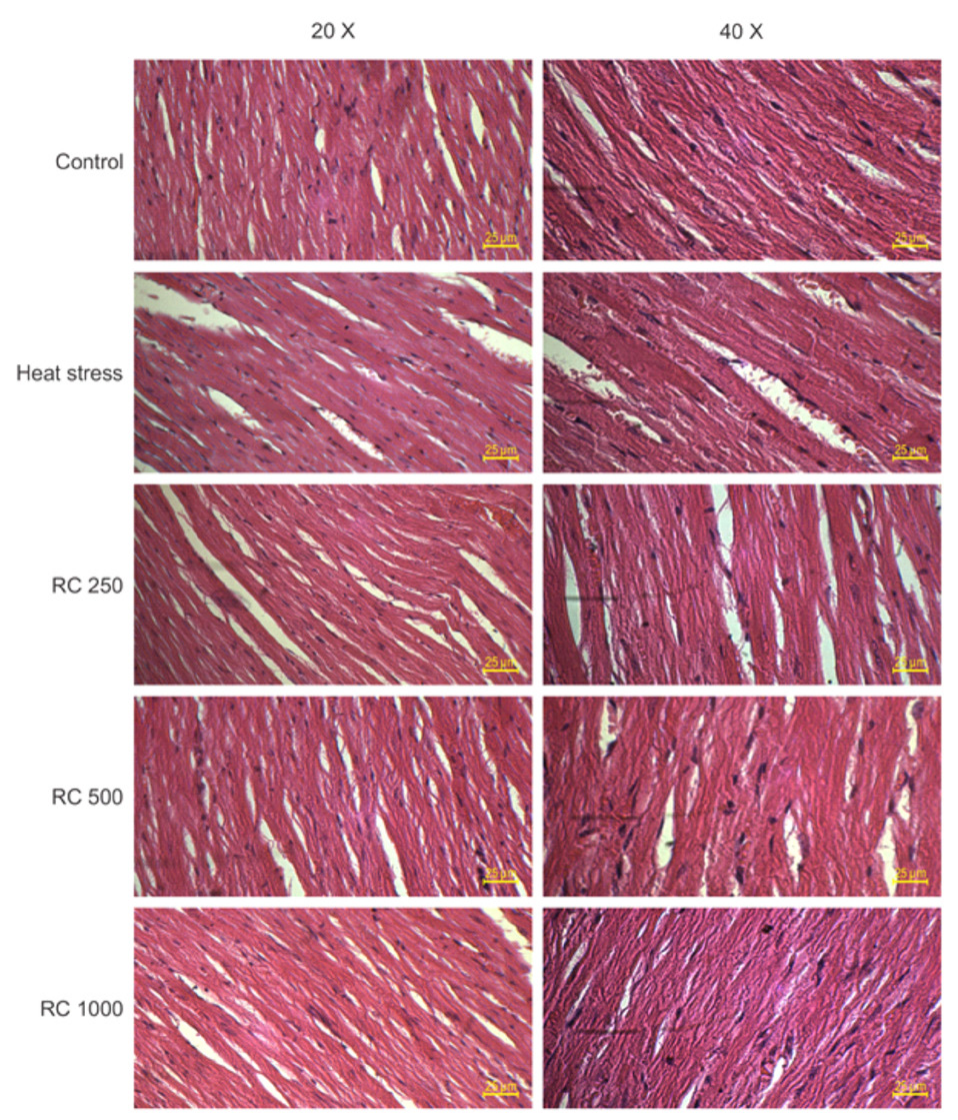
Hematoxylin and eosin (H&E) staining of the heat stress exposed animals. Rc: rosacanina.

The results of the histopathological changes of heart tissues showed that normothermic control group rats had a normal myofibrillar structure and activity of myocytes (Figure 5). In the heat-stressed rat hearts, enlarged myocardial cells along with extended space between muscle fibers and shrunken nuclei, a disordered arrangement of cells and loss of striations transverse, degeneration of cardiomyocytes, increased amount of inflammatory cell, and increased capillary blood flow were seen. All these changes were improved by RC administrations at doses of 500 and 1000 mg/mL.

## Discussion

The main findings of the current experiment revealed that pretreatment with different doses of RC decreased heat stress-induced excessive ROS production and inhibited PERK/eIF2α/CHOP signaling and caspase 8 activation. Further, our findings showed that RC inhibited apoptosis in heat-stressed rats, evidenced by a decreased activation of caspase 8 and diminished myocardial apoptotic cell death.

Cardiovascular dysfunction and myocardial damage are common extreme heat exposure complications, but the potential underlying mechanisms are not clear.^31,32^ Accumulating evidence emphasizes the role of oxidative stress or the accumulation of ROS and ER stress in cardiovascular diseases.^33-37^ This study indicated that chronic heat stress-induced oxidative damage in the heart tissue as reflected by increased ROS levels. The previous studies consistently reported that heat stress increases free radicals and diminishes the antioxidant defense system in the heart tissue.^14,38,39^ These findings raise the possibility that persistent ER stress induced by heat stress led to the accumulation of UP in the ER lumen provoking Ca^2+^ leakage and excessive ROS production.^37^ Additionally, evidence shows that excessive ROS generation and thereby oxidative stress not only induce ER stress but also are consequences of ER stress.^40^ Therefore, ROS production can be the upstream initiator or downstream effector of ER stress. Nevertheless, pretreatment with RC at doses of 500 and 1000 mg/mL markedly attenuated ROS overproduction in left ventricular samples in heat stress-exposed rats. Evidence has proven the antioxidant property of RC, which is mainly attributed to its high phenolic and vitamin C contents.^41-43^ This study showed the protective effect of RC against heat stress-induced oxidative damage in the heart tissue.

During ER stress, activated PERK phosphorylates its target protein eIF2α, suppressing protein synthesis lessen the accumulation of UP.^44^ In this study, heat stress resulted in the activation of ER transmembrane protein kinases, including PERK and eIF2α, and activated CHOP protein. This result is in line with the results of Yang et al. reporting that heat stress led to the ER stress in the myocardium.^14^ Several studies have reported that heat stress can activate the ER stress response in different tissues.^45,46^ Nevertheless, pretreatment with RC markedly decreased phosphorylation of PERK and eIF2α and protein levels of CHOP, suggesting that RC attenuated ER stress in the myocardium.

Furthermore, several lines of studies have reported that heat stress can promote cell death in cardiomyocytes, which may contribute to cardiac dysfunction.^8,47,48^ In contrast to extreme temperatures exposure (49-50 °C) which causes rapid necrotic cell death^49^, intense heat stress (41-43 ^°^C) can accelerate apoptosis cell death.^50,51^ In this study, the results of the TUNEL assay presented supportive information for biochemical analyses and showed that heat stress (43 °C for 15 min/day) induced apoptosis cell death. Also, in the current study, heat stroke-induced cardiac injury in rats. The data showed that administering RC ameliorated myocardial histopathological changes caused by heat stress. In agreement with our results, previous studies have revealed that heat shock can lead to heart damage, whereas treatment with pharmacological and non-pharmacological agents have reversed the heat stress-induced myocardial injury.^14,15^

Apoptosis is a caspase-dependent programmed form of cell death.^52^ Caspase 8 is the main initiator caspase in the extrinsic pathway of apoptosis, which cleaves and activates downstream caspases such as caspase 3. Moreover, it can activate the intrinsic apoptosis pathway mediated by mitochondria.^53^ In the present study, chronic heat stress could activate caspase 8 in the cardiomyocytes, which were accompanied by an enhanced number of TUNEL-positive cells. In support of our results, several studies have also demonstrated the importance of caspase-8 in heat stress-induced apoptosis.^54,55^

In contrast, some in vitro studies have shown that acute heat stress could not induce caspase 8 activation.^56,57^ On the other hand, RC pretreatment at doses of 500 and 1000 mg/mL could decrease caspase 8 activation as well as the number of TUNEL-positive cells in the cardiomyocytes of heat-stressed rats. These results suggest a cardioprotective effect of RC in heat stress conditions.

CHOP plays a pro-apoptotic role in the process of stress transmission. It has been demonstrated that the CHOP-mediated apoptosis pathway is implicated in the process of cell death under physiological and pathological conditions.^58^ Several studies have shown that under prolonged and severe ER stress conditions, UPR can activate apoptosis pathways by activation of CHOP protein.^44,59^ Moreover, previous studies have demonstrated that increased CHOP protein expression mediates ER stress-associated apoptosis while lack of CHOP expression results in a resistance to ER stress-induced apoptosis.^60,61^ Similarly, we found that two weeks of heat stress exposure led to overexpression of CHOP protein accompanied by an increased number of apoptotic cell death in the cardiac tissue, attenuated by RC treatments. It seems that RC, through inactivation of PERK and eIF2α, inhibited CHOP activation and ER stress-mediated apoptosis.

## Conclusion

**Figure 6:**
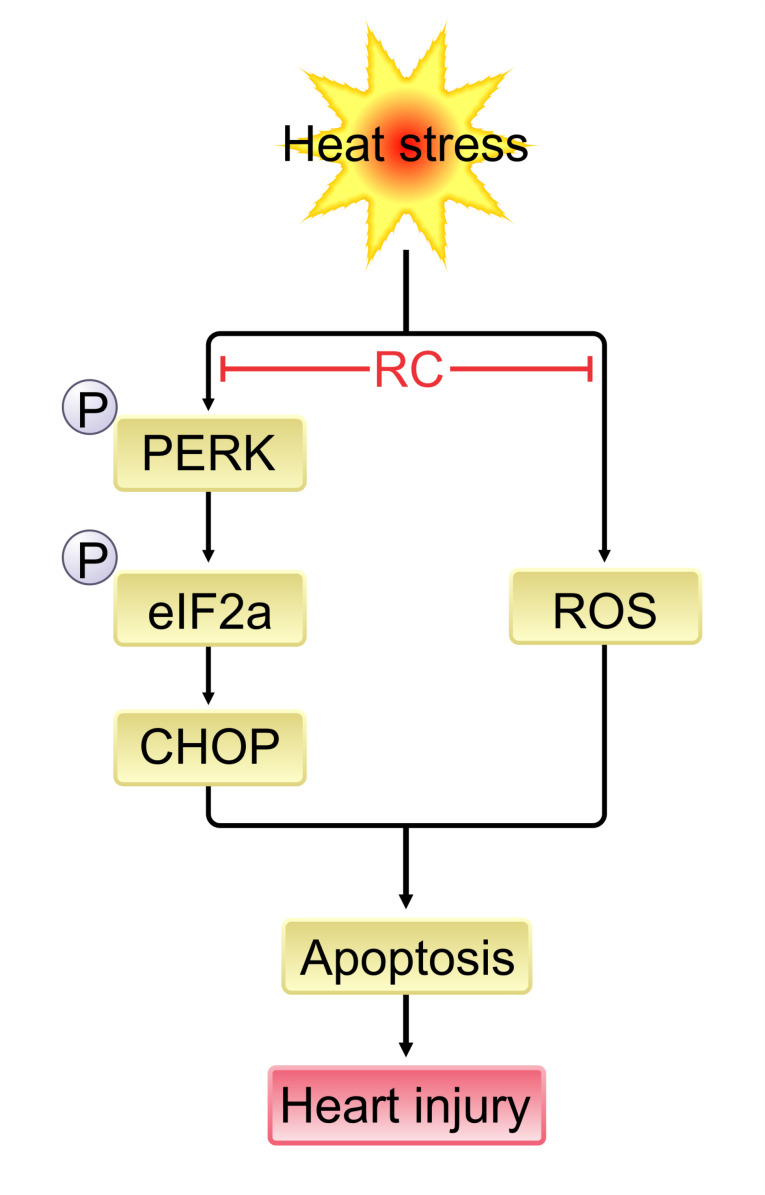
Schematic model of the protective effect of *Rosa canina* against heat stress injury

In summary, the findings of the present study indicated that attenuation of ROS levels, ER stress, and myocardial apoptosis are the possible mechanisms underlying cardioprotective effects of RC against heat stress. Therefore, pretreatment with RC may provide a preventive approach to lessen cardiovascular dysfunction induced by exposure to heat stress (Figure 6).

## Acknowledgments

The authors thank the staff of Neurosciences Research Center (NSRC) for it support and assistance with this project.

## Competing interests

The authors declared no conflict of interest.

## Ethical approval

All experimental procedures were carried out according to the National Research Council (US) Committee for the Update of the Guide for the Care and Use of Laboratory Animals (Guide for the Care and Use of Laboratory Animals, 8th edition, 2011), and confirmed by the Higher Academic Education Institute of Rab-Rashid IACUC Committee (Code number: 96/4981/14).

## Funding

None.
